# Temporal dynamics of resting EEG networks are associated with prosociality

**DOI:** 10.1038/s41598-020-69999-5

**Published:** 2020-08-03

**Authors:** Bastian Schiller, Tobias Kleinert, Sarah Teige-Mocigemba, Karl Christoph Klauer, Markus Heinrichs

**Affiliations:** 1 Department of Psychology, Laboratory for Biological and Personality Psychology, University of Freiburg, Stefan-Meier-Straße 8, 79104 Freiburg, Germany; 2 Freiburg Brain Imaging Center, University Medical Center, University of Freiburg, Freiburg, 79104 Germany; 3 Department of Psychological Diagnostics, Philipps-University of Marburg, Marburg, 35032 Germany; 4 Department of Psychology, Social Psychology and Methodology, University of Freiburg, Freiburg, 79085 Germany

**Keywords:** Human behaviour, Social neuroscience, Cooperation, Psychology

## Abstract

As prosociality is key to facing many of our societies’ global challenges (such as fighting a global pandemic), we need to better understand why some individuals are more prosocial than others. The present study takes a neural trait approach, examining whether the temporal dynamics of resting EEG networks are associated with inter-individual differences in prosociality. In two experimental sessions, we collected 55 healthy males’ resting EEG, their self-reported prosocial concern and values, and their incentivized prosocial behavior across different reward domains (money, time) and social contexts (collective, individual). By means of EEG microstate analysis we identified the temporal coverage of four canonical resting networks (microstates A, B, C, and D) and their mutual communication in order to examine their association with an aggregated index of prosociality. Participants with a higher coverage of microstate A and more transitions from microstate C to A were more prosocial. Our study demonstrates that temporal dynamics of intrinsic brain networks can be linked to complex social behavior. On the basis of previous findings on links of microstate A with sensory processing, our findings suggest that participants with a tendency to engage in bottom-up processing during rest behave more prosocially than others.

## Introduction

Prosocial behavior, such as cooperating, sharing resources, and providing aid, is widespread, universal^1,2^ and, as shown recently, of critical importance to solve global issues like fighting a pandemic^3^. However, substantial inter-individual differences exist in the extent to which these behaviors are shown^4–11^. While some individuals voluntarily risk their own lives by, for instance, helping others to handle Covid-19, others put their own interests first and break rules established to protect other (high risk) individuals. Recent research has shown that prosocial behavior in different incentivized economic game paradigms is correlated, as is this behavior and self-reported prosociality, pointing to the existence of a domain-general “prosocial phenotype”^12–14^. To illuminate heterogeneity in this prosocial phenotype, it could be useful to rely on a so-called neural trait approach which has already identified objective and stable neural sources (e.g., brain volume or resting state activity) of individual differences in various other phenotypes^15–17^. As a key advantage, neural traits are free of any biases (e.g., social desirability, self-deception) inherent in self-report measures^18^. Relying on this approach, we here use a spatio-temporal analysis of multichannel electroencephalography^19,20^ recorded at rest to investigate whether temporal dynamics of intrinsic large-scale brain networks are linked with prosociality.

Our study adds to previous studies using the neural trait approach to predict prosociality^21,22^ in four regards. First, we analyze changes in scalp electrical potential topographies, thereby considering changes in global network activity. Second, using these networks’ intrinsically generated activity, we attempt to identify associations of brain activity and prosocial behavior independent from specific social contexts. Third, we uniquely illuminate the association of the temporal dynamics of neural resting networks with prosocial behavior on a millisecond scale. And fourth, we investigate a domain-general prosocial phenotype^14,23^ across distinct measurement approaches (self-report and incentivized behavior), social contexts (individual and collective), and reward domains (money and time).

More specifically, we used a spatio-temporal analysis approach to cluster the resting EEG signal into a circumscribed number of scalp electrical potential topographies that remain stable for certain time periods (ca. 50–120 ms) before dynamically changing into a different topography that remains stable again^24–28^. One has referred to these periods with stable topographies as “microstates” and one has interpreted transitions between microstates to represent sequential coordinated activity of different, distributed neural networks. Remarkably, almost 80% of the variance in the resting EEG data can be explained by just four archetypal microstates A–D, i.e., resting networks which may result from evolutionarily determined, brain-intrinsic biases toward particular patterns of co-activation particularly suited to representing environmentally relevant information^29^. These networks’ temporal dynamics have been proven to be highly reliable, specific, and reproducible across multiple independent studies^30,31^, ideally qualifying them as neural trait markers.

Several studies have attempted to identify the neural sources and functions related to these four resting EEG networks^20,32,33^. Microstates A and B have been associated with bottom-up sensory processing (microstate A has been linked to activity in temporal areas involved in phonological processing and microstate B has been linked to activity in extrastriate areas involved in visuo-spatial processing^32–35^). Microstate D, on the other hand, has been associated with top-down cognitive processing (microstate D has been linked to activity in fronto-parietal areas involved in attention and control^32,34,36^). The function of microstate C has remained more controversial, as it was originally linked to activity in fronto-insular areas considered to be involved in salience processing^34^, but recent research associates it with activity in the default mode network and stimulus-independent processing^20,37^.

Being the first study of its kind, our general research question was to examine whether there is any association among the four canonical resting EEG networks’ temporal dynamics with prosociality. For that purpose, we generated a domain-general index of prosociality that we aggregated across self-reported prosocial concern (using the Interpersonal Reactivity Index^38^ scale Empathic Concern), self-reported prosocial values (using the Portrait Value Questionnaire^39^ scale Benevolence), collective prosocial behavior (using the Public Goods Game^40^ in a monetary reward domain), and individual prosocial behavior (using the Social Value Orientation task^41^ in a non-monetary reward domain). On the basis of resting EEG networks’ significance for non-social behavior, personality, and psychiatric conditions^20,31^, we expected that revealing an individual’s tendency to engage these networks at rest would help explain inter-individual differences in prosociality and illuminate the potential psychological processes that underlie these differences. For example, our findings might contribute to the debate on the role of bottom-up and top-down processing in driving inter-individual differences in prosocial behavior^42–45^. Due to our study’s exploratory nature, we applied Bonferroni-correction for multiple tests to our findings.

## Results

### Prosociality

**Figure 1:**
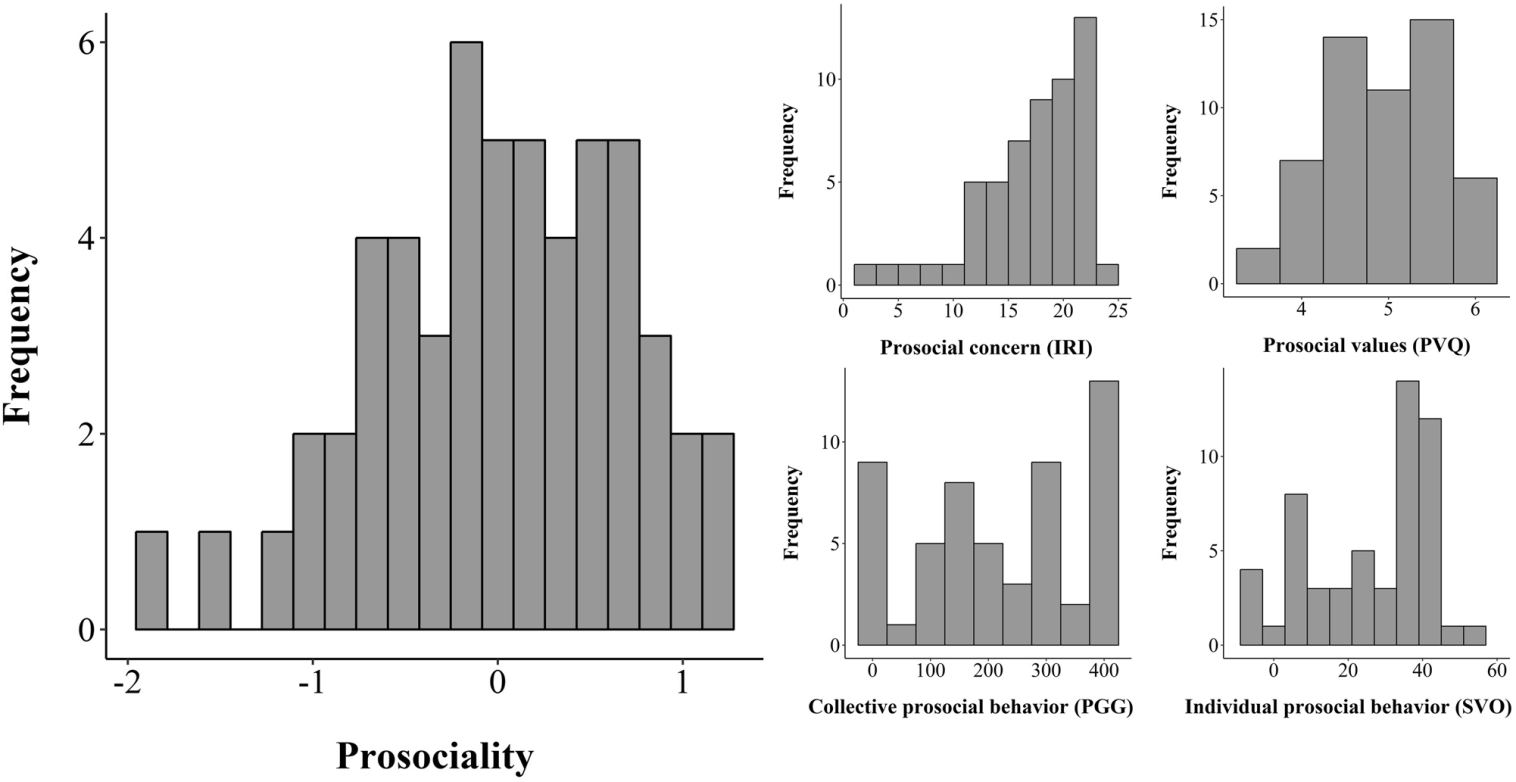
Variability in prosociality. Left: Histogram indicating significant variability in the aggregated prosociality index which we generated relying on research demonstrating the existence of a domain-general “prosocial phenotype”^12–14^. Right: Histograms indicating significant variability in prosocial concern (middle top; values of the Interpersonal Reactivity Index scale Empathic Concern), prosocial values (right top; values of the Portrait Value Questionnaire scale Benevolence), collective prosocial behavior (middle bottom; monetary contributions in the Public Goods Game), and individual prosocial behavior (right bottom; Social Value Orientation angles distributing time units); note that all these variables were entered into our calculation of the domain-general prosociality index.

We did observe considerable variability in prosociality (*M* = 0.00, s.d. = 0.69, range: − 1.84–1.23), aggregated across self-reported prosocial concern (non-standardized values in the 7-item 5-point Likert scale Empathic Concern scale of the IRI: *M* = 17.63, s.d. = 4.62, range: 2–24), self-reported prosocial values (non-standardized values in the 6-point Likert scale Benevolence of the PVQ: *M* = 4.90, s.d. = 0.67, range: 3.50–6.00), collective prosocial behavior (non-standardized contributions in the Public Goods Game: *M* = 220.00, s.d. = 142.27, range: 0–400), and individual prosocial behavior (non-standardized angles in the Social Value Orientation task: *M* = 26.20, s.d. = 16.71, range: − 7.82–53.41; for histograms displaying the distributions of all variables’ non-standardized values, see Fig. 1).

### Prosociality and resting EEG networks

**Figure 2:**
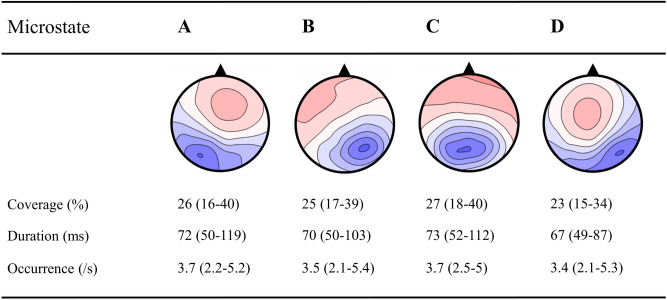
Topographies of grand-mean microstates with descriptive statistics. Grand mean maps for *N* = 55 participants, their percentage coverage, mean duration in millisecond, and mean occurrences per second. Note that the four empirically identified microstates closely resemble the canonical resting-state microstates^20^.

In accordance with previous findings, the applied cluster analysis identified the four canonical microstates A–D (see Fig. 2) which explained 78% of the variance in our whole sample and at least 69% of the variance in every single individual (*M* = 77.73, s.d. = 3.50, range: 69.07–84.19). To assess reliability, we correlated microstate parameters that were identified separately for the first and second halves of artifact-free data available in each individual (for details, see “Methods”). We detected correlations ranging from 0.772–0.922 (all *P* < 0.001) with regard to microstate coverage, duration, and occurrence, and correlations ranging from 0.281 to 0.720 (all *P* < 0.038) concerning microstate transitions (for details, see Table S2). These findings show the potential of microstate parameters to serve as neural trait markers that might be associated with inter-individual differences in prosociality.

**Figure 3:**
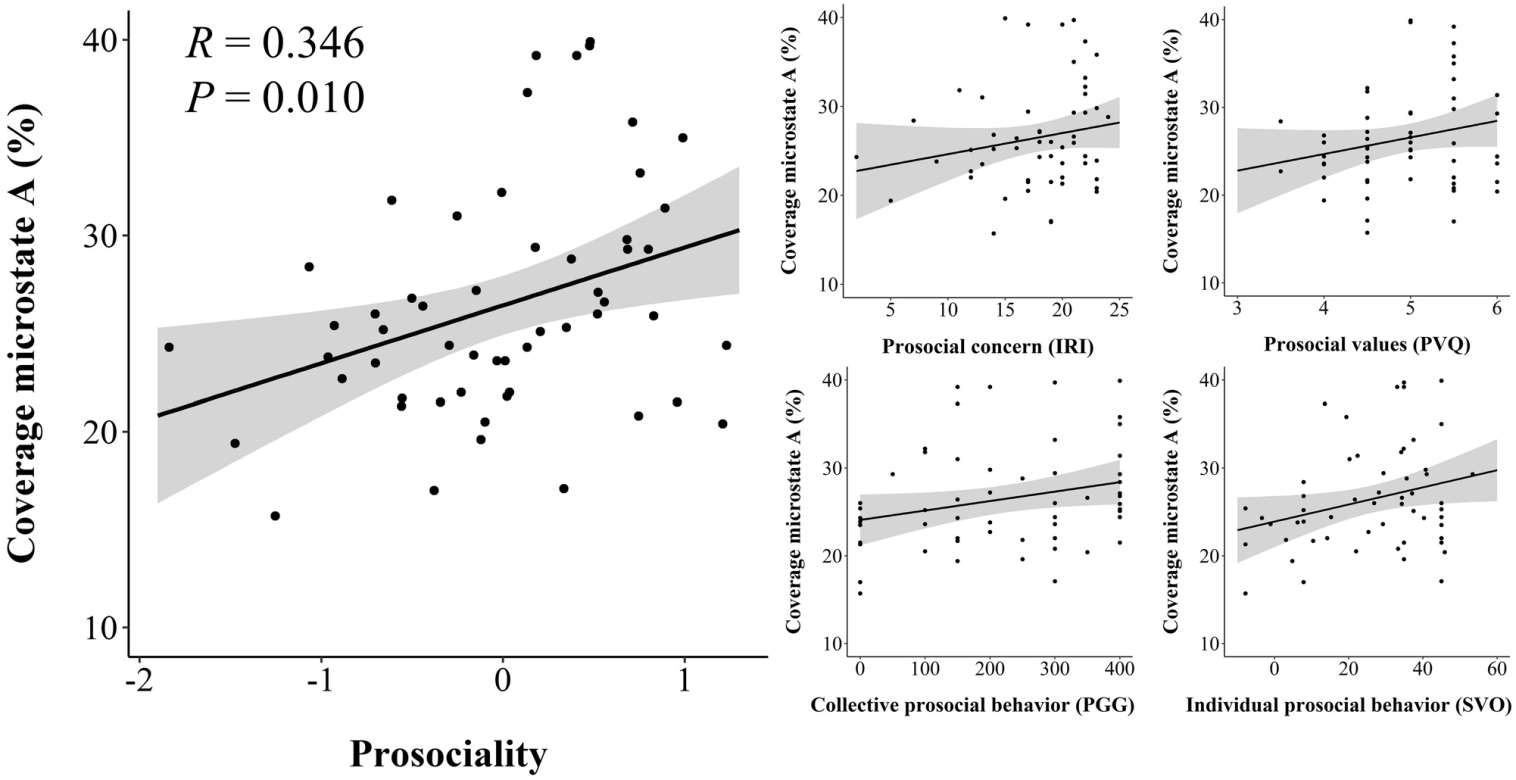
Associations of the coverage of microstate A with prosociality. Left: Scatterplot of the association of the coverage of microstate A (in % of total time) with the prosociality index including 95% confidence intervals. Right: Scatterplots of the associations of the coverage of microstate A with prosocial concern (middle top; values of the Interpersonal Reactivity Index scale Empathic Concern), prosocial values (right top; values of the Portrait Value Questionnaire scale Benevolence), collective prosocial behavior (middle bottom; monetary contributions in the Public Goods Game), and individual prosocial behavior (right bottom; Social Value Orientation angles distributing time-units) including 95% confidence intervals.

We first tested for associations of microstate coverage and prosociality. Correlative analyses revealed that participants with a higher coverage of microstate A were more prosocial [*R*_*s*_(53) = 0.346, *P* = 0.010, significant after Bonferroni-correction for multiple testing; prosocial concern: *R*_*s*_(53) = 0.192, *P* = 0.159; prosocial values: *R*_*s*_(53) = 0.165, *P* = 0.230; individual prosocial behavior: *R*_*s*_(53) = 0.284, *P* = 0.035; collective prosocial behavior: *R*_*s*_(53) = 0.213, *P* = 0.118, see Fig. 3]. Meng’s z-tests indicated that the correlation of prosociality with the coverage of microstate A was significantly higher than the correlation of prosociality with the coverage of microstates B [*R*_*s*_(53)_Prosociality × Coverage B_ = − 0.190, *Z*(53) = 2.514, *P* = 0.006)], C [*R*(53)_Prosociality × Coverage C_ = 0.012, *Z*(53) = 1.914, *P* = 0.028] and D [*R*(53)_Prosociality × Coverage D_ = − 0.265, *Z*(53) = 2.526, *P* = 0.006]. Subsequent analyses revealed that the association of prosociality with the coverage of microstate A was mainly due to an association of prosociality with microstate A’s duration [*R*_*s*_(53) = 0.370, *P* = 0.005], and not its occurrence [*R*(53) = 0.097, *P* > 0.20]. We also found a marginally significant negative correlation between the coverage of microstate D and prosociality [*R*(53) = -0.265, *p* = 0.051]. There were no other significant associations of microstate coverages and prosociality (see Table S3). Furthermore, we found the significant association of microstate A’s coverage and prosociality in both the first and the second half of data (first half: *R*(53) = 0.301, *P* = 0.025; second half: *R*(53) = 0.375, *P* = 0.005), demonstrating the robustness of this finding. In sum, participants with a higher temporal stability (= mean duration) and, in turn, coverage of microstate A tended to show more prosocial concern, values, and behavior.

**Figure 4:**
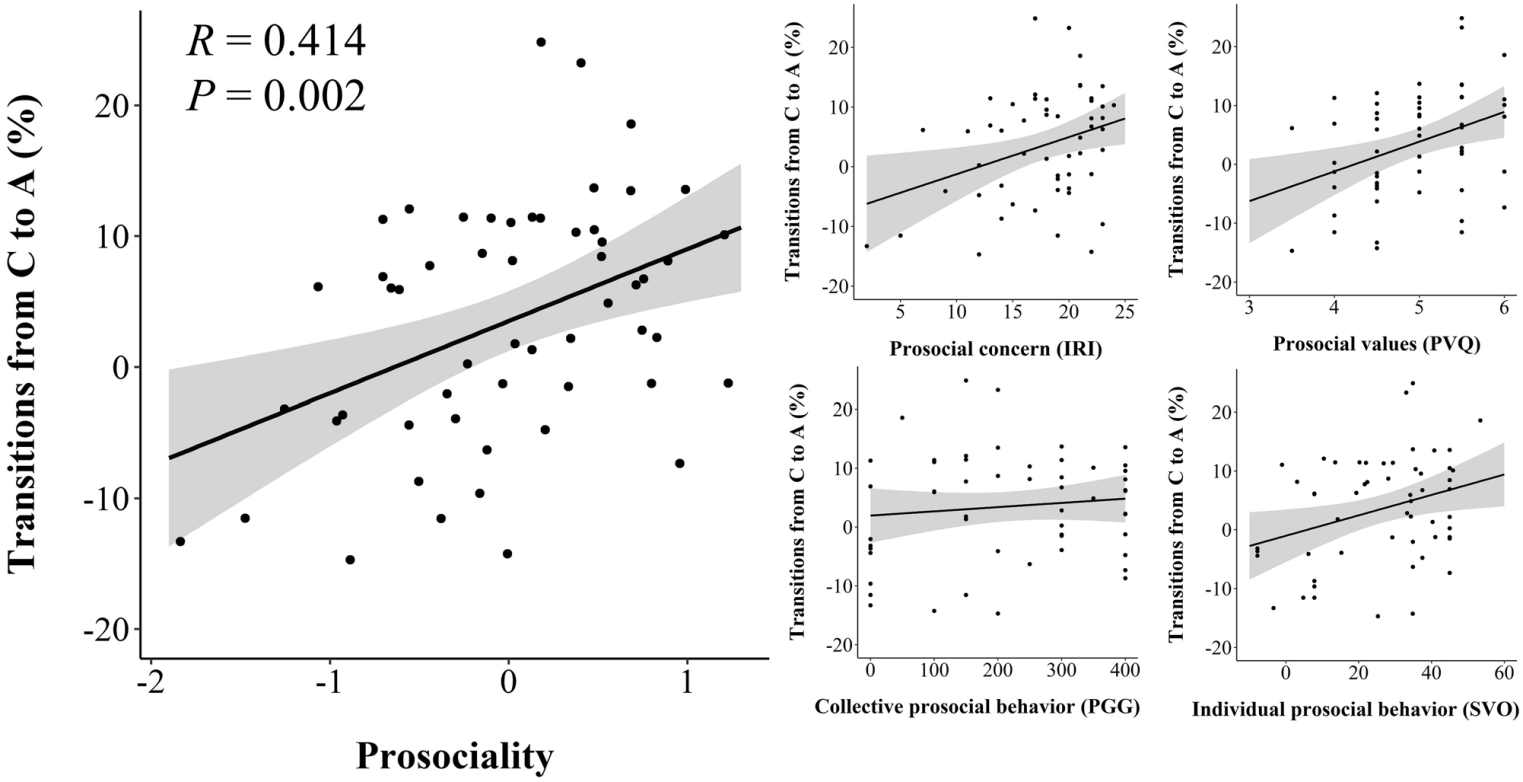
Associations of transitions from microstate C to A with prosociality. Left: Scatterplot of the association of transitions from microstate C to A (visualized is the percentage of observed transitions relative to expected transitions; i.e., a value of 10 indicates that transitions from microstate C to A occurred 10% more frequently than expected from each microstate’s occurrence) with the prosociality index including 95% confidence intervals. Right: Scatterplots of the associations of transitions from microstate C to A with prosocial concern (middle top; values of the Interpersonal Reactivity Index scale Empathic Concern), prosocial values (right top; values of the Portrait Value Questionnaire scale Benevolence), collective prosocial behavior (middle bottom; monetary contributions in the Public Goods Game), and individual prosocial behavior (right bottom; Social Value Orientation angles distributing time-units) including 95% confidence intervals.

Next we tested for associations of microstate transitions and prosociality. Correlative analyses revealed that participants with more transitions from microstate C to A were more prosocial [*R*(53) = 0.414, *P* = 0.002, significant after Bonferroni-correction for multiple testing; prosocial concern: *R*_*s*_(53) = 0.261, *P* = 0.054; prosocial values: *R*_*s*_(53) = 0.366, *P* = 0.006; collective prosocial behavior: *R*_*s*_(53) = 0.081, *P* > 0.20; individual prosocial behavior: *R*_*s*_(53) = 0.261, *P* = 0.054, see Fig. 4]. There were no other significant associations of microstate transitions and prosociality after Bonferroni-correction for multiple testing (see Table S3). Meng’s z-tests indicated that the correlation of prosociality with transitions from microstate C to A was significantly higher than prosociality’s correlations with all other transition types (all *P* < 0.049) except for transitions from microstates D to A [*R*_*s*_(53)_Prosociality × Transition D to A_ = 0.154, *Z*(53) = 1.52, *P* = 0.064] and A to C [*R*(53)_Prosociality × Transition A to C_ = 0.332, *Z*(53) = 0.632, *P* = 0.264]. Furthermore, we found the significant association of transitions from microstate C to A and prosociality in both the first and the second half of data [first half: *R*_*s*_(53) = 0.374, *P* = 0.005; second half: *R*(53) = 0.316, *P* = 0.019], demonstrating the robustness of this finding. In sum, participants with more transitions from microstate C to A tended to show more prosocial concern, values, and behavior.

Finally, to enable better comparability of our findings with the literature, we repeated all of our analysis sorting individual microstate maps according to normative grand mean templates^29^. These analyses yielded highly similar results confirming the robustness of our study’s findings [correlation of coverage of microstate A and prosociality: *R*(53) = 0.345, *P* = 0.010; correlation of transitions from microstate C to A and prosociality: *R*(53) = 0.268, *P* = 0.048; for details see Table S3].

## Discussion

Can someone’s task-free neurophysiological processing reveal information on someone’s prosociality? By analyzing the spatio-temporal dynamics of resting EEG recordings the present study demonstrates that an individual’s propensity of how to engage the four canonical EEG resting networks (i.e., microstates A, B, C, and D) is associated with an index of an individual’s domain-general prosociality. This index was aggregated across self-reported prosocial concern and values, as well as incentivized behavior collected in different reward domains (time and money) and social contexts (individual and collective). More specifically, we found that participants with a higher coverage of microstate A and more transitions from microstate C to A were more prosocial.

How can we interpret the association of microstate A’s coverage with prosociality based on previous research examining the functional significance of the four canonical EEG resting networks (for reviews, see^20,31^)? In healthy participants, microstate A’s coverage has been associated with activity in the phonological fMRI resting network^34^ and been shown to increase during hypnosis—a state characterized by a sense of automaticity and effortlessness^46,47^. One could thus deduce from these findings that a higher coverage of microstate A indicates an individual’s tendency to engage in sensory, bottom-up processing during rest which in turn predisposes towards prosociality. Notably, microstate B, associated with visual sensory processing^34,37^ but also shown to decrease during hypnosis^47^, was not associated with prosociality, indicating a complex relationship between different kinds of sensory, bottom-up processing and prosociality. Furthermore, we found indications of a negative association of attention-related microstate D^34,37,48^ with prosociality (this correlation was no longer significant after correction for multiple comparisons across microstate classes), tentatively suggesting an antagonistic relationship of specific kinds of top-down compared to bottom-up processing with prosociality which may be corroborated in future research.

Our study also demonstrates that inter-individual differences in prosociality are associated with differences in the communication between EEG resting networks. More specifically, the networks underlying microstates A and C seem to act as a crucial gateway, as demonstrated by positive associations of transitions from microstate C to A with prosociality. So far, no consensus has been reached about the function of microstate C, but recent research has proposed that this state is associated with stimulus-independent processing^20,37,49^. One could thus speculate that individuals with more transitions from microstate C to A tend to shift more often from stimulus-independent processing to stimulus-dependent sensory-related, bottom-up processing which in turn seems to predispose them to prosociality. We encourage future research to zoom in on the transitions between resting EEG microstates in order to better understand the functions underlying the communication between neural resting networks on a millisecond scale^20,31^.

The discussed findings and interpretations might also contribute to our understanding of the interactive role of bottom-up and top-down processing in driving inter-individual differences in prosocial behavior^42–44^. It has been reported that individuals who self-report a bottom-up processing style tend to maintain more successful interpersonal relationships, exhibit greater prosocial concerns, and tend to behave more prosocially^50–52^. Other research both examining and manipulating the response times underlying social behavior showed that individuals who took or possessed less time for their decisions behaved more prosocially^53,54^ (but see also^55,56^). These findings have been interpreted to indicate that people are “intuitively cooperative” and behave prosocially if they do not take or have the time to engage in time-consuming top-down processing *during* the (decision) task (see also recent research for specification under which conditions these findings hold^57–59^). Here, we complement these findings by demonstrating correlative evidence that a tendency to engage in bottom-up rather than top-down, *task-free* neural processing predisposes towards prosociality.

While prosociality is clearly determined by situational factors as well^60,61^, recent research has demonstrated that there is a cross-situational and temporally stable individual tendency for prosociality^14,62^. The present study proposes potential neural traits affecting this tendency by uniquely revealing the temporal dynamics of resting EEG networks with millisecond resolution, thereby extending findings from research on the role of resting fMRI networks in prosociality^63,64^. It shows that an individual’s propensity of how to engage the four resting EEG networks is associated with an individual’s level of prosociality. Future studies should test the generalizability of these findings across situations, as the aforementioned associations seemed to differ in their significance and strength across distinct measurement approaches, social contexts, and reward domains. Furthermore, given that males and females differ in their prosociality in several dimensions^65–67^, it is unclear how our findings would apply to females. We hope that our study inspires future research aiming to better understand the nature of the relationship between resting EEG networks’ temporal dynamics and prosociality in participants of both genders, for example by experimentally modifying the environment of resting EEG recordings^68^, analyzing the relationship between task-independent and task-dependent neural activity^16,69,70^, analyzing brain-to-brain synchronization affecting prosociality^71^, and linking the four resting EEG networks to psychological constructs known to affect prosociality (e.g. empathy, perspective-taking^72–74^).

## Methods

### Participants

Based on an estimated average medium effect size of associations between resting EEG networks’ temporal dynamics and trait variables in similar research^75^, 55 participants (*α* = 0.05, *β* = 0.85) are needed to detect a significant effect (Correlation, bivariate normal model; G-Power^76^). Our sample included 55 healthy, right-handed participants (*M* = 24.22 y, s.d. = 4.20, range: 19–40 y). Due to potential confounds associated with hormonal variation in the menstrual cycle and the complexities associated with controlling for this variation in the experimental design^62,63^, only male participants were enrolled in this initial project. All participants were right-handed, and free of current or previous history of physical and psychiatric disorders, and alcohol or drug abuse. The Ethics Committee of the University of Freiburg approved this study, which was conducted according to the principles expressed in the Declaration of Helsinki.

### Procedure

There were two experimental sessions. At the first session, participants received detailed information on the experiment and gave informed consent. Then, participants were comfortably seated in a darkened, electrically shielded cabin for the recording of 64-channel resting EEG. Our measurement protocol consisted of 20-s eyes open periods followed by 40-s eyes closed periods, repeated five times. This resting state paradigm has been routinely used in resting EEG research^16,17,27,77^ in order to minimize fluctuations in participants’ vigilance state. Participants can become drowsy already after 3 min of recording resting state brain activity, if there is no alternation of eyes-open/eyes-closed periods^78^. We gave the instructions about eye opening/closing via intercom. To exclude the possibility that instruction delivery confounds the resting state during the eyes-closed periods, instructions were delivered at the beginning and end of the eyes-open periods. After the resting EEG measurement participants completed three reaction time paradigms that are unrelated to the purpose of the current study and will be analyzed elsewhere. Finally, participants completed self-report measures related to prosociality. The first session lasted approximately 1.5 h. The second session was conducted in groups of six participants in a group-laboratory specifically designed for computerized interaction experiments several weeks after the first appointment. After receiving detailed instructions and answering comprehension questions, participants played two social-decision making paradigms. Finally, participants had to complete reaction time tasks which they had to repeat several times dependent on the time units earned in one of the social decision-making paradigms; we did not analyze this task further. The second session lasted approximately 1.5 h. After the experiment, subjects received an average compensation of 45.13 € (s.d. = 1.18, range: 43.50–48.10), depending on participants’ and their interaction partners’ decisions.

### EEG recording

The EEG was recorded with a 64-channel recording system (Brainamp with actiCAP, Brain Products Gmbh, Munich) according to the extended 10–20 system montage^79^. Scalp impedance was kept below 10 kΩ. FCz served as the reference electrode, AFz as the ground electrode. Horizontal and vertical electrooculographic signals were recorded with two additional electrodes at the left and right outer canthi and one electrode at the left infraorbital. The EEG was online band-pass filtered between 0.1 and 100 Hz, and the data digitized with a sampling rate of 500 Hz.

### EEG pre-processing

We used the Brain Vision Analyzer program (Version 2.1.0.327; Brain Products GmbH, Munich) to pre-process EEG data. Only the 200 s eyes-closed periods were used for the analysis, because the influence of external visual stimulus processing and confounding eye blinks is minimized^80,81^. Next, we band-pass filtered EEG data (high-pass 2 Hz, low-pass 20 Hz^29^ and re-derived them to average reference. Ocular correction was conducted via a semi-automatic independent component analysis based correction process. EEG signals with excessive noise were replaced by using a linear interpolation of neighboring electrodes. After an automatic artifact rejection (maximum amplitude: ± 100 μV), data were visually examined to eliminate residual artifacts. Finally, and in line with previous research, artifact-free data (*M* = 166.91 s, s.d. = 30.41 s, range: 56–200 s) were segmented into 2-s epochs for further analyses^16,17,82^.

### EEG microstate analysis

Microstate analysis was conducted using the microstate-plugin^83^ for the Matlab Toolbox EEGLAB^84^. First, in line with the standard procedure^19,85^, the maps at the momentary peaks of the Global Field Power (i.e., maximum voltage values at all electrodes that represent time points of optimal signal-to-noise ratio^29^were extracted and submitted to a modified spatial cluster analysis using the atomize-agglomerate hierarchical clustering method (AAHC^86,87^). This clustering approach identified the four most dominant cluster maps in every single participant. The individual cluster maps were submitted to a second cluster analysis yielding grand mean maps which were then sorted according to the standard labeling of the four canonical EEG resting networks^20,31^ (see Fig. 2). Next, the maps of each individual were sorted according to these grand mean maps on the basis of spatial correlations. To enable comparability of our findings with the literature, we repeated this sorting procedure using the grand mean template maps as identified in normative EEG data collected from multiple sites (n = 496; 19 electrodes^29^. Finally, the GFP peaks of individual EEG data were assigned to the individually identified cluster maps to which they best fitted. This assignment was linearly interpolated to the time periods between the GFP peaks, yielding a continuous temporal stream of microstates occurring in each individual. From this last step, we extracted several microstate parameters. We focused on the temporal *coverage* a given microstate is dominant, representing the total presence of the underlying network. We also investigated underlying associations with average microstate *duration* (i.e., an index of the temporal stability of the underlying network; unit: milliseconds) and microstate *occurrence* (i.e., an index of the relative usage of the underlying network; unit: occurrences/second), both of which together determine a microstate’s temporal *coverage*. To reveal communication between the underlying networks, we finally studied microstate *transitions,* which were operationalized as the percentage of observed transitions from one microstate class to another relative to expected transitions [transitions = (observed transitions per second – expected transitions per second) / expected transitions per second * 100; i.e., a value of 10 indicates that transitions from one microstate class to another occurred 10% more frequently than expected from each microstate’s occurrence]. Finally, to assess the reliability of microstate parameters, we performed the fitting procedure (i.e., assigning the GFP peaks to the four individually identified maps, which were sorted according to the grand mean maps or grand mean templates maps) separately for the first and second halves of each participant’s artifact-free EEG data.

### Self-report measures of prosocial concern and prosocial values

We measured self-reported prosocial concern using the *Interpersonal Reactivity Index* (IRI^38^, German version by^88^). The IRI is a questionnaire including 28 items for the assessment of empathic abilities on four different scales with seven items each (Empathic Concern, Perspective Taking, Fantasy, and Emotional Distress). Per item, participants have to report how well these items describe them as a person from “not at all” to “very strong” (5-point Likert scale). For the purpose of this study, we focused on the scale *Empathic Concern* which assesses feelings of prosocial concern for others (Cronbach’s Alpha = 0.68–0.73^89^). Additionally, we measured self-reported prosocial values using the Portrait Value Questionnaire (PVQ^90^, German version by^39^). We used the 21-item version of the PVQ measuring personal values on 10 different scales with 2–3 items each (Benevolence, Power, Achievement, Hedonism, Stimulation, Self-direction, Universalism, Tradition, Conformity, and Security). The PVQ measures values indirectly by obtaining judgments of the similarity of another person, who is portrayed in terms of her or his goals, aspirations, and wishes, to oneself, on a scale ranging from “not like me at all” to “very much like me” (6-point Likert scale). For the purpose of this study, we focused on the scale *Benevolence*, which assesses prosocial values, i.e., attaching importance to the preservation and enhancement of the welfare of other people (Cronbach’s Alpha = 0.67^90^).

### Decision-making paradigms to assess prosocial behavior

We measured prosocial behavior involving actual consequences to interaction partners across two distinct reward domains (money and time) and social contexts (individual and collective). To control for strategic considerations (e.g., reputation, reciprocity), we kept the decisions anonymous and non-reciprocal. Because we were interested in explaining inter-individual differences, we used a fixed order of the two decision-making paradigms^17,91^. Participants first played the *Public Goods Game*, a well-established behavioral measure of collective prosocial behavior^92^. Each participant was randomly assigned to a group of three people and endowed with 400 points (exchange rate: 100 points = 1 Euro) which he could either keep for himself or contribute to the public good (possible contributions ranged from 0 to 400 in steps of 50 points). The sum of all contributions to the public good was multiplied by the factor 1.5 and then equally divided among all players of the assigned group regardless of individual contributions. This game induces a conflict between self and group interests, because participants can earn the maximal personal profit by contributing nothing and profiting from other participants’ contributions, whereas the group can earn the maximal profit by all participants contributing all their resources. The amount contributed to the public good is entered into the calculation of domain-general *Prosociality*. Second, participants played the Social Value Orientation task^41^, which assesses individual prosocial behavior and has previously been linked to prosocial behavior such as helping or pro-environmental intentions^93,94^. In our study, two participants who were explicitly not aligned to the same group in the Public Goods Game were randomly paired for the SVO. Both participants had to decide how to distribute time units between themselves and another participant among a series of six preset choices which affected the total duration of the experiment for each participant (see Fig. S1 for an exemplary item of the SVO; 100 points = 2 min; for another experiment using time units, see^95^). After the decisions it was randomly determined which of the two paired participant’s decisions were implemented into actual consequences. This task induces a conflict between self and another person’s interests, because participants can earn the maximal personal profit in terms of time units by selecting a distribution which does not yield the maximal profit for both paired participants and vice versa. From all these decisions an SVO angle is calculated which represents a continuous measure of how much weight someone attaches to the welfare of others in relation to their own. The SVO angle is also entered into the calculation of domain-general Prosociality (see Statistical Analysis,see Fig. S1 for details on the calculation of the SVO angle).

### Statistical analysis

To obtain a domain-general measure of prosociality we averaged z-standardized scores of self-reported prosocial concern and prosocial values, and of PGG contributions and SVO angles (Cronbach’s Alpha = 0.62). To test for any association between specific microstates’ *coverages* and prosociality and for the reliabilities of microstates parameters (first vs. second half), Pearson-coefficients were calculated for normally distributed variables, and Spearman-coefficients otherwise (2-sided tests, alpha level = 0.05, see Table S1). We only report findings significant after Bonferroni-correction for multiple testing across microstates (four tests; *P* < 0.0125) and the transitions between them (twelve tests; *P* < 0.00417). In case of significant associations of a microstate/microstate transition and prosociality, we tested for this finding’s specificity by comparing it with the associations of the other microstates/microstate transitions and prosociality using Meng’s z-tests for dependent correlations^96^.

## Supplementary information

Supplementary information

## Data Availability

All data generated during and/or analyzed during the current study are available from the corresponding authors on reasonable request.
