# Supplementary material for: Temporal dynamics of resting EEG networks are associated with prosociality
Source: Sci Rep. 2020 Aug 3;10:13066. doi: 10.1038/s41598-020-69999-5 (PMC7400630; doi:10.1038/s41598-020-69999-5)
Supplement: Supplementary file 1 — Supplementary information [file 41598_2020_69999_MOESM1_ESM.docx]

Supplemental Material

Temporal dynamics of resting EEG networks are associated with prosociality

Bastian Schiller*^1,2^, Tobias Kleinert^1^, Sarah Teige-Mocigemba^3^, Karl Christoph Klauer^4^, and Markus Heinrichs*^1,2^

^1^Department of Psychology, Laboratory for Biological and Personality Psychology, Albert-Ludwigs University of Freiburg, Stefan-Meier-Straße 8, 79104 Freiburg, Germany

^2^Freiburg Brain Imaging Center, University Medical Center, Albert-Ludwigs University of Freiburg, Freiburg, Germany

^3^Department of Psychological Diagnostics, Philipps-University of Marburg, Marburg, Germany

^4^Department of Psychology, Social Psychology and Methodology, Albert-Ludwigs University of Freiburg, Freiburg, Germany

***Corresponding authors**

Dr. phil. Bastian Schiller and Prof. Dr. Markus Heinrichs

Department of Psychology, Laboratory for Biological and Personality Psychology

Albert-Ludwigs-University of Freiburg

Stefan-Meier-Strasse 8

D-79104 Freiburg i. Br. Germany

Mail: [schiller@psychologie.uni-freiburg.de](mailto:schiller@psychologie.uni-freiburg.de); [heinrichs@psychologie.uni-freiburg.de](mailto:heinrichs@psychologie.uni-freiburg.de)

Fax: +49-(0)761 203-3023

Phone: +49-(0)761 203-3029


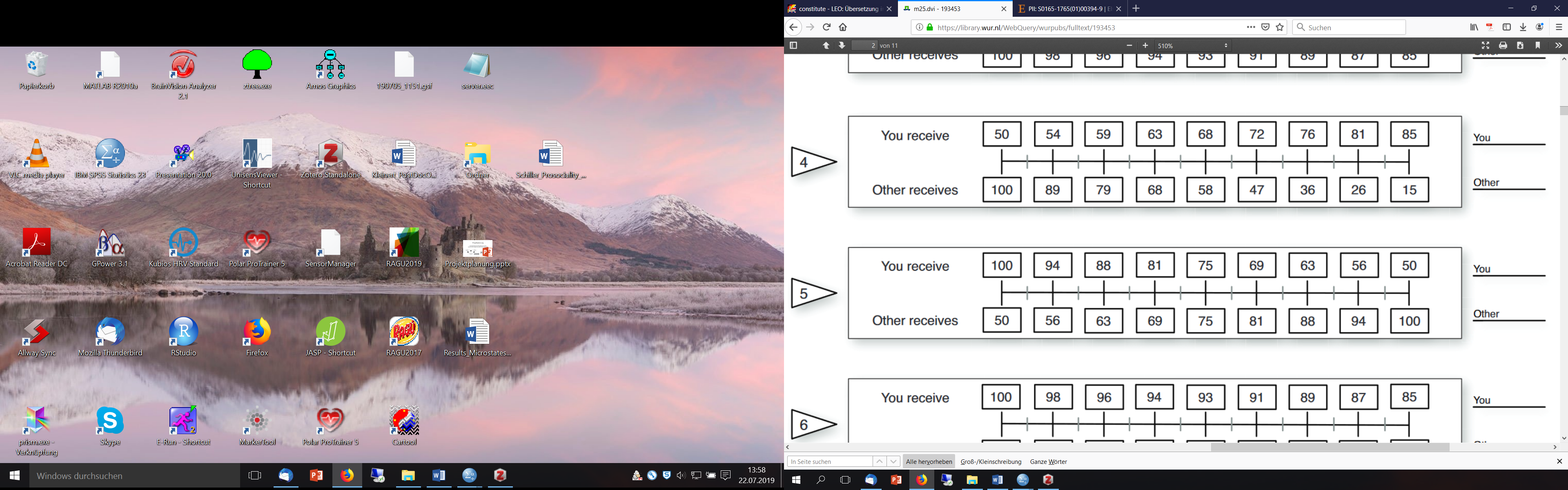


**Figure S1** Exemplary item from the SVO slider measure.

Based on six items, the SVO yields a final score in the form of an angle in a two-dimensional space with the dimensions payoff to self and payoff to other. A low value represents a competitive (< -12.04°; Maximizing own payoff and minimizing other’s payoff) or an individualistic orientation (-12.04° to 22.45°; Maximizing own payoff with no regard to other’s payoff), while a higher value represents a prosocial (22.45° to 57.15°; avoiding inequality) or an altruistic orientation (> 57.15°; Maximizing other’s payoff with no regard to own payoff). In the presented item (picture from Murphy *et al.*, 2011) the left choice (own payoff = 100, other’s payoff = 50) represents an individualistic decision, the middle choice (own payoff = 75, other’s payoff = 75) a prosocial decision, and the right choice (own payoff = 50, other’s payoff = 100) an altruistic decision. A competitive decision is not available in this item (e.g., own payoff = 90, other’s payoff = 25). In our study, the SVO-angle is used as a metric indicator of prosocial behavior.

**Table S1** Descriptive statistics and normality tests

| **Variable** | **Min** | **Max** | **Mean** | **s.d.** | **Shapiro-Wilk test (P-value)** |
| --- | --- | --- | --- | --- | --- |
| **Measures of prosociality** | | | | | |
| Prosociality (domain general) | -1.84 | 1.23 | 0 | 0.69 | 0.543 |
| Collective prosocial behavior (PGG) | 0 | 400 | 220 | 142.27 | 0.000 |
| Individual prosocial behavior (SVO) | -7.82 | 53.41 | 26.20 | 16.71 | 0.002 |
| Prosocial concern (IRI) | 2 | 24 | 17.69 | 4.86 | 0.000 |
| Prosocial values (PVQ) | 3.50 | 6 | 4.94 | 0.67 | 0.003 |
| **Microstate parameters** | | | | | |
| Coverage A | 15.70 | 39.90 | 26.45 | 5.91 | 0.016 |
| Coverage B | 16.60 | 39.00 | 24.49 | 5.36 | 0.020 |
| Coverage C | 17.60 | 40.10 | 26.56 | 5.06 | 0.161 |
| Coverage D | 14.90 | 32.70 | 22.50 | 4.03 | 0.239 |
| Duration A | 50.40 | 119 | 72.09 | 12.96 | 0.015 |
| Duration B | 50.40 | 103 | 70.06 | 11.75 | 0.185 |
| Duration C | 52.10 | 112 | 72.75 | 14.29 | 0.019 |
| Duration D | 49.10 | 86.70 | 66.64 | 9.80 | 0.173 |
| Occurrence A | 2.24 | 5.22 | 3.70 | 0.69 | 0.655 |
| Occurrence B | 2.10 | 5.43 | 3.53 | 0.69 | 0.810 |
| Occurrence C | 2.53 | 5.04 | 3.70 | 0.59 | 0.615 |
| Occurrence D | 2.11 | 5.29 | 3.43 | 0.71 | 0.337 |
| Transition A to B | -34.59 | 24.35 | -5.37 | 11.21 | 0.251 |
| Transition A to C | -11.89 | 26.70 | 5.05 | 8.13 | 0.945 |
| Transition A to D | -48.64 | 23.51 | -2.10 | 11.72 | 0.251 |
| Transition B to A | -39.03 | 28.60 | -3.03 | 12.30 | 0.006 |
| Transition B to C | -32.14 | 17.21 | 0.581 | 9.17 | 0.060 |
| Transition B to D | -18.37 | 18.09 | 0.671 | 8.33 | 0.849 |
| Transition C to A | -14.69 | 24.83 | 3.53 | 9.19 | 0.392 |
| Transition C to B | -39.95 | 23.56 | 0.300 | 11.36 | 0.177 |
| Transition C to D | -39.74 | 25.19 | -6.31 | 12.11 | 0.306 |
| Transition D to A | -45.99 | 29.35 | 0.93 | 11.69 | 0.006 |
| Transition D to B | -24.01 | 22.28 | 1.38 | 9.42 | 0.856 |
| Transition D to C | -36.96 | 29.73 | -4.20 | 11.60 | 0.616 |
| Template-sorting: Coverage A | 15.70 | 40.60 | 26.26 | 6.00 | 0.282 |
| Template-sorting: Duration A | 50.40 | 110.00 | 72.38 | 12.74 | 0.017 |
| Template-sorting: Occurrence A | 2.24 | 5.22 | 3.66 | 0.751 | 0.664 |
| Template-sorting: Transition C to A | -32.15 | 22.36 | 0.49 | 9.78 | 0.311 |

*N* = 55. Descriptive statistics (minimum, maximum, mean, standard deviation) and normality tests (Shapiro Wilk tests, significant in case of *P* < 0.05) for all variables in the study.

**Table S2** Reliabilities of microstate parameters (first vs. second half of resting EEG measurement)

| **Microstate parameters** | ***R/R****_s_* | ***P*** |
| --- | --- | --- |
| Coverage A | 0.817^s^ | < 0.001 |
| Coverage B | 0.869^s^ | < 0.001 |
| Coverage C | 0.786 | < 0.001 |
| Coverage D | 0.772 | < 0.001 |
| Duration A | 0.864^s^ | < 0.001 |
| Duration B | 0.913^s^ | < 0.001 |
| Duration C | 0.832^s^ | < 0.001 |
| Duration D | 0.922 | < 0.001 |
| Occurrence A | 0.911 | < 0.001 |
| Occurrence B | 0.888 | < 0.001 |
| Occurrence C | 0.861 | < 0.001 |
| Occurrence D | 0.870 | < 0.001 |
| Transition A to B | 0.546^s^ | < 0.001 |
| Transition A to C | 0.281^s^ | 0.038 |
| Transition A to D | 0.567^s^ | < 0.001 |
| Transition B to A | 0.720 | < 0.001 |
| Transition B to C | 0.506^s^ | < 0.001 |
| Transition B to D | 0.430 | 0.001 |
| Transition C to A | 0.674 | < 0.001 |
| Transition C to B | 0.671^s^ | < 0.001 |
| Transition C to D | 0.702 | < 0.001 |
| Transition D to A | 0.669 | < 0.001 |
| Transition D to B | 0.474 | < 0.001 |
| Transition D to C | 0.633 | < 0.001 |
| Template-sorting: Coverage A | 0.878 | < 0.001 |
| Template-sorting: Duration A | 0.806^s^ | < 0.001 |
| Template-sorting: Occurrence A | 0.925 | < 0.001 |
| Template-sorting: Transition C to A | 0.554^s^ | < 0.001 |

*N* = 55. Reliabilities of microstate parameters as calculated by correlations of parameters obtained from the first half of the individual resting EEG measurement with parameters obtained from the second half of the individual resting EEG measurement. Pearson correlation coefficients were used in case of normally distributed variables, and Spearman correlation coefficients otherwise (indicated by superscript letters).

**Table S3** Correlations of microstate parameters with prosociality

|  | **Prosociality** | | **Prosocial concern (IRI)** | | **Prosocial values (PVQ)** | | **Collective prosocial behavior (PGG)** | | **Individual prosocial behavior (SVO)** | |
| --- | --- | --- | --- | --- | --- | --- | --- | --- | --- | --- |
| **Microstate parameters** | ***R/R****_s_* | ***P*** | ***R/R****_s_* | ***P*** | ***R/R****_s_* | ***P*** | ***R/R****_s_* | ***P*** | ***R/R****_s_* | ***P*** |
| Coverage A | **0.346** | **0.010** | 0.192 | 0.159 | 0.165 | 0.230 | 0.284 | 0.035 | 0.213 | 0.118 |
| Coverage B | -0.190 | 0.165 | -0.053 | 0.698 | -0.303 | 0.024 | -0.066 | 0.632 | -0.123 | 0.370 |
| Coverage C | 0.012 | 0.933 | -0.067 | 0.626 | 0.041 | 0.766 | -0.116 | 0.397 | -0.016 | 0.905 |
| Coverage D | -0.265 | 0.051 | -0.180 | 0.190 | -0.082 | 0.550 | -0.185 | 0.176 | -0.171 | 0.213 |
| Duration A | **0.370** | **0.005** | 0.096 | 0.485 | 0.355 | 0.008 | 0.181 | 0.185 | 0.285 | 0.035 |
| Duration B | 0.078 | 0.573 | 0.026 | 0.851 | 0.098 | 0.475 | 0.027 | 0.847 | 0.082 | 0.553 |
| Duration C | 0.183 | 0.181 | -0.012 | 0.928 | 0.248 | 0.068 | 0.093 | 0.500 | 0.157 | 0.252 |
| Duration D | 0.059 | 0.671 | -0.066 | 0.630 | 0.236 | 0.082 | -0.027 | 0.845 | 0.110 | 0.425 |
| Occurrence A | 0.097 | 0.482 | 0.095 | 0.492 | -0.121 | 0.377 | 0.074 | 0.590 | -0.048 | 0.730 |
| Occurrence B | -0.269 | 0.047 | -0.069 | 0.618 | -0.406 | 0.002 | -0.086 | 0.535 | -0.164 | 0.231 |
| Occurrence C | -0.232 | 0.089 | -0.024 | 0.861 | -0.183 | 0.182 | -0.173 | 0.208 | -0.209 | 0.126 |
| Occurrence D | -0.254 | 0.061 | -0.130 | 0.345 | -0.208 | 0.128 | -0.096 | 0.486 | -0.231 | 0.090 |
| Transition A to B | -0.109 | 0.426 | -0.057 | 0.679 | -0.238 | 0.080 | -0.012 | 0.928 | -0.011 | 0.934 |
| Transition A to C | 0.332 | 0.013 | 0.245 | 0.072 | 0.262 | 0.053 | 0.050 | 0.716 | 0.330 | 0.014 |
| Transition A to D | -0.170 | 0.215 | -0.160 | 0.244 | 0.114 | 0.409 | 0.013 | 0.924 | -0.255 | 0.060 |
| Transition B to A | 0.071 | 0.607 | 0.037 | 0.791 | 0.058 | 0.673 | 0.059 | 0.678 | 0.039 | 0.779 |
| Transition B to C | -0.210 | 0.123 | -0.134 | 0.329 | -0.070 | 0.611 | -0.057 | 0.678 | -0.234 | 0.085 |
| Transition B to D | 0.051 | 0.713 | 0.117 | 0.395 | -0.007 | 0.958 | -0.082 | 0.554 | 0.143 | 0.296 |
| Transition C to A | **0.414** | **0.002** | 0.267 | 0.049 | 0.366 | 0.006 | 0.081 | 0.559 | 0.261 | 0.054 |
| Transition C to B | -0.192 | 0.160 | -0.081 | 0.557 | -0.283 | 0.036 | 0.078 | 0.573 | -0.210 | 0.124 |
| Transition C to D | -0.188 | 0.170 | -0.139 | 0.312 | -0.098 | 0.478 | -0.216 | 0.113 | -0.025 | 0.857 |
| Transition D to A | 0.154 | 0.262 | 0.060 | 0.664 | -0.067 | 0.628 | 0.316 | 0.019 | 0.005 | 0.972 |
| Transition D to B | 0.121 | 0.379 | 0.064 | 0.645 | 0.155 | 0.258 | -0.074 | 0.592 | 0.240 | 0.078 |
| Transition D to C | -0.187 | 0.172 | -0.124 | 0.368 | -0.055 | 0.688 | -0.282 | 0.037 | -0.122 | 0.374 |
| Template-sorting: Coverage A | **0.345** | **0.010** | 0.161 | 0.241 | 0.277 | 0.041 | 0.133 | 0.332 | 0.248 | 0.068 |
| Template-sorting: Duration A | **0.342** | **0.011** | 0.051 | 0.711 | 0.399 | 0.003 | 0.147 | 0.286 | 0.283 | 0.036 |
| Template-sorting: Occurrence A | 0.068 | 0.619 | 0.070 | 0.610 | -0.045 | 0.744 | 0.024 | 0.860 | 0.001 | 0.995 |
| Template-sorting: Transition C to A | 0.268 | 0.048 | 0.136 | 0.324 | 0.316 | 0.019 | 0.218 | 0.109 | 0.253 | 0.063 |

*N* = 55. Correlations of prosociality with microstate parameters. Pearson correlation coefficients were used in case of normally distributed variables (see Table S1), and Spearman correlation coefficients otherwise. Bold printed values indicate significant correlations of prosociality with microstate features after Bonferroni-correction for multiple testing (in case of coverage, duration and occurrence of microstates we corrected for 4 tests, in case of transitions of microstates we corrected for 12 tests).
